# Oral Squamous Cell Carcinoma Associated with Dental Implants: A Literature Review with Focus on Field-Cancerized Mucosa

**DOI:** 10.3390/cancers18010017

**Published:** 2025-12-19

**Authors:** Maria Cuevas-Nunez, Cosimo Galletti, Gianluca Tenore, Umberto Romeo, Rosa Ballester Victoria, María José Biosca Gómez de Tejada, Javier Bara-Casaus, Maria Teresa Fernández Figueras

**Affiliations:** 1 College of Dentistry, Universitat Internacional de Catalunya, C/de Josep Trueta, s/n, 08195 Barcelona, Spain; 2 Department of Oral and Maxillofacial Surgery, Hospital Univeritari Mutua Terrassa, 08221 Barcelona, Spain; jbara@mutuaterrassa.cat; 3 Faculty of Medicine and Surgery, University of Enna, 94100 Enna, Italy; 4 Department of Oral Sciences and Maxillofacial Surgery, Sapienza University of Rome, 00161 Rome, Italy; 5 Pathology, Hospital Universitari General de Catalunya, 08195 Barcelona, Spain; 6 Oral and Maxillofacial Surger, Hospital Universitari General de Catalunya, 08195 Barcelona, Spain; 7 Medicina, Facultad de Medicina y Ciencias de la Salud, UIC Barcelona International University of Catalonia, 08195 Barcelona, Spain

**Keywords:** oral squamous cell carcinoma, dental implants, oral field cancerization

## Abstract

Background: The oncologic safety of dental implants (DIs) in patients with oral field cancerization (OFC) remains uncertain. Increasing reports of oral squamous cell carcinoma (OSCC) developing adjacent to DIs have raised concerns regarding the interaction between implants, chronic inflammation, and genetically altered mucosa. Methods: A comprehensive PubMed/MEDLINE search was performed through June 2025 to identify English- or Spanish-language publications reporting primary OSCC occurring in proximity to DIs. Extracted variables included patient demographics, tumor site, clinical presentation, presence of oral potentially malignant disorders (OPMD) or prior OSCC, peri-implant inflammation, management, and outcomes. Cases describing clinical features compatible with field-altered mucosa (e.g., OPMD or prior OSCC) were evaluated descriptively, recognizing that formal histopathologic or molecular evidence of OFC was rarely reported. Results: A total of 105 implant-associated OSCC cases were identified. The mean patient age was 66.8 years (range: 40–90), with a female predominance (1.3:1). The mandible was the most frequently involved site (86.7%). A prior history of OPMD or OSCC was reported in 53 patients (50.5%), and peri-implantitis preceding diagnosis in 21 cases (19.0%). The most common clinical presentations were exophytic (59.0%) and ulcerated (37.1%) lesions, frequently mimicking peri-implantitis and contributing to diagnostic delay. Reported outcomes included recurrence in 11 cases (10.5%) and death in 13 cases (12.4%). Conclusions: Current evidence suggests that implant-associated OSCC frequently occurs in patients with pre-existing mucosal alterations and may be influenced by the interaction of field cancerization with local inflammatory and mechanical factors. Implant rehabilitation in individuals with OPMD, prior OSCC, or epithelial dysplasia should be undertaken cautiously, with rigorous long-term surveillance to ensure oncologic safety.

## Simple Summary

Dental implants are widely used to replace missing teeth and are generally considered safe. Recently, reports have described oral cancers developing near implants. Many of these cancers occurred in people who already had potentially malignant or malignant changes in the mouth lining, a condition known as field cancerization, where the tissue is genetically more vulnerable to developing cancer. To better understand this issue, we reviewed all published cases of oral cancer that appeared next to dental implants. Many patients had a history of potentially risky oral conditions, and early signs of cancer were sometimes mistaken for simple inflammation around the implant. Although these findings do not show that implants cause cancer, they indicate that implant placement and follow-up may warrant additional attentiveness in individuals with field-altered tissues. Careful evaluation of unusual changes and long-term monitoring could support earlier recognition of potential problems.

## 1. Introduction

Oral squamous cell carcinoma (OSCC) is the most common malignancy affecting the oral mucosa, accounting for approximately 90% of all oral cancers worldwide [1]. Despite advances in diagnosis and therapy, OSCC continues to present significant clinical challenges, with an overall five-year survival rate of 50–60%. This low survival rate is largely attributed to delayed diagnosis and the high incidence of second primary tumors. The etiology of OSCC is multifactorial, involving well-established carcinogens such as tobacco, alcohol, and, in a subset of cases, infection with high-risk human papillomavirus (HPV) [2,3,4]. Other factors, including genetic predisposition, immunosuppression, poor oral hygiene, and chronic inflammation, have been proposed as additional cofactors [5].

In the past three decades, dental implants (DI) have become an essential part of modern restorative dentistry, providing durable functional and esthetic rehabilitation. As implant use expands among aging populations, new biological concerns have emerged, including reports of OSCC arising adjacent to osseointegrated implants [6]. Although the number of such cases is small relative to the vast number of implants placed globally, their occurrence has raised critical questions regarding possible etiopathogenic links between implant-related inflammation and carcinogenesis [7,8].

Peri-implantitis, a chronic inflammatory condition characterized by progressive bone loss and mucosal destruction around implants, has been frequently documented in patients subsequently diagnosed with OSCC [9]. While this observation does not imply causation, it has prompted hypotheses that chronic peri-implant inflammation could act as a local promoting factor in genetically susceptible mucosa. One proposed mechanism involves the release of metallic particles or ions from titanium surfaces through corrosion or mechanical wear, triggering oxidative stress and persistent inflammation [9]. This proinflammatory environment may alter epithelial behavior, favoring DNA damage and the activation of oncogenic pathways.

In many reported cases, patients with implant-associated OSCC also had traditional carcinogenic risk factors (tobacco, alcohol) [10] or a history of oral potentially malignant disorders (OPMD) such as leukoplakia, erythroplakia, proliferative verrucous leukoplakia (PVL), or oral lichen planus (OLP) [11]. These findings support the concept that implant-associated OSCC often arises within a “field cancerized” mucosa, an area of epithelium with widespread genetic or epigenetic alterations, predisposing it to multifocal carcinogenesis [12].

The concept of oral field cancerization (OFC), introduced by Slaughter in 1953, describes the phenomenon whereby large areas of oral epithelium exhibit precancerous molecular changes even before clinically visible lesions appear [12]. These fields may remain quiescent for years but retain malignant potential, explaining the occurrence of multiple synchronous or metachronous primary tumors. The interaction between OFC and dental implants is particularly relevant because implants are typically placed in alveolar mucosa, a site not commonly associated with de novo OSCC in non-implanted patients. Thus, their presence may influence the local behavior of genetically unstable mucosa or exacerbate carcinogenic processes through chronic inflammatory stimuli.

Clinically, the early presentation of OSCC near implants often mimics peri-implant disease. Swelling, erythema, and ulcerations are common features shared by both conditions, leading to frequent misdiagnosis and therapeutic delay [13]. As a result, some lesions are only identified after persistent non-healing or during surgical exploration. Given these overlapping features, it is essential that clinicians maintain a high index of suspicion for malignancy in cases of atypical or refractory peri-implant inflammation.

In view of these diagnostic and pathogenic uncertainties, the oncologic safety of dental implants in patients with a history of OFC, OPMD, or previous OSCC remains unclear. A systematic evaluation of available evidence is warranted to better understand the frequency, clinical features, and outcomes of these cases.

Therefore, this scoping review aimed to (1) summarize the demographic, clinical, and pathologic characteristics of OSCC associated with dental implants, and (2) critically examine the possible role of oral field cancerization in their pathogenesis and recurrence patterns.

## 2. Materials and Methods

### 2.1. Study Design

**Figure 1:**
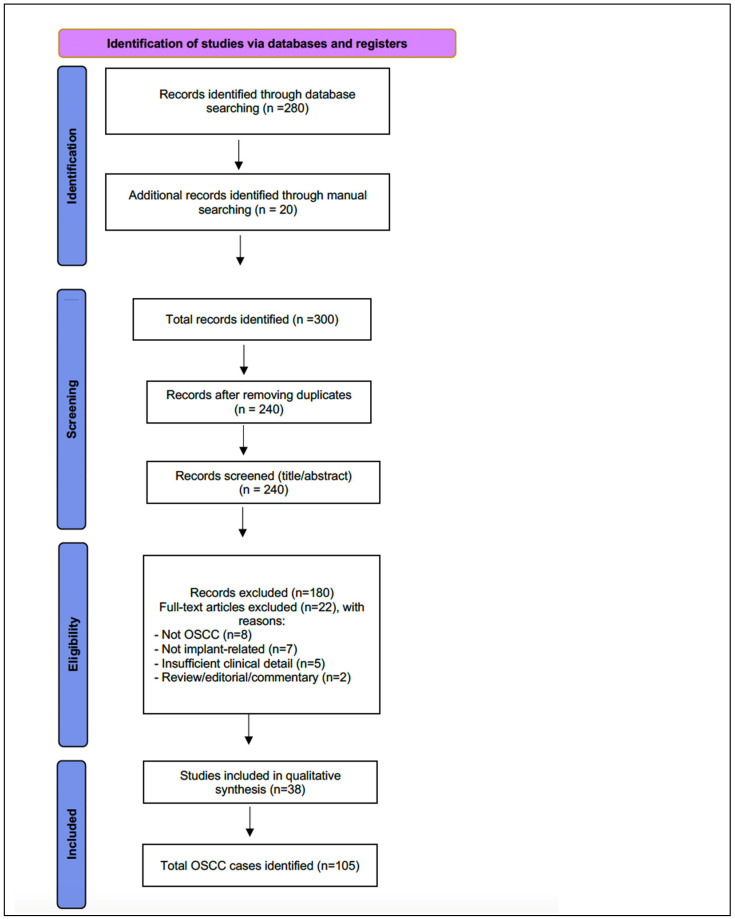
PRISMA-style flow diagram of the study identification and screening process.

This study consisted of a scoping review of the literature on OSCC associated with DI. The objective was to describe demographic and clinicopathologic characteristics of reported cases, as well as to evaluate potential etiologic considerations. The review adhered to PRISMA-ScR recommendations for reporting scoping reviews. Because this was not a registered systematic review, a PRISMA-style flow diagram (Figure 1) is provided descriptively to enhance transparency regarding study identification and screening.

### 2.2. Literature Review

A comprehensive primary search was conducted in PubMed/MEDLINE, supplemented by a Google Scholar search to capture potentially relevant gray literature and case reports not indexed in traditional databases. The following search strategy was used: (“oral squamous cell carcinoma” OR “OSCC”) AND (“dental implant” OR “peri-implantitis” OR “osseointegrated implant”). The search was limited to studies published in English or Spanish. Titles and abstracts were screened to identify studies reporting primary OSCC arising adjacent to one or more DI. Inclusion criteria were: (1) histologic confirmation of OSCC; (2) description of implant involvement or location; and (3) availability of patient-level clinical data. Exclusion criteria included review articles without new case data, non-SCC malignancies, and animal or in vitro studies. The full texts of eligible studies were reviewed independently by two authors (MCC and CG). Disagreements were resolved through discussion and consensus. No formal risk-of-bias assessment was undertaken, which is consistent with scoping reviews based primarily on case reports and case series.

For each included case, the following data were extracted: patient demographics, risk factors (e.g., tobacco, alcohol use), presence of periimplantitis, OPMD or carcinoma, implant location, clinical presentation, treatment, outcome and follow up. Data on implant material, surface characteristics, prosthetic design, loading protocols, OPMD subtype, and epithelial dysplasia were also extracted when explicitly reported, although these variables were inconsistently documented and therefore could not be consistently analyzed.

### 2.3. Data Analysis

Descriptive statistics were used to summarize demographic and clinical features of published cases. Frequencies, percentages, and mean values were calculated for relevant variables. Owing to the heterogeneity and incomplete reporting across studies, no inferential statistical analyses were performed. All analyses were conducted using Microsoft Excel (version 16.101.3; Microsoft Corporation, Redmond, WA, USA).

## 3. Results

A total of 105 published cases of OSCC associated with DI were identified between 1983 and 2024. These cases originated from 38 different studies, which to our knowledge represents one of the most comprehensive datasets reported to date. To minimize the risk of double counting, all cases were cross-checked across studies by comparing demographic and clinical variables. To the best of our assessment, no duplicate cases were identified. A complete summary of the published cases, including demographic information, clinical presentation, and outcomes, is presented in Supplementary Table S1.

### 3.1. Demographics

The mean age of affected patients was 66.8 years (range: 40–90). A slight female predominance 60/105 (56.2%) was observed. Male patients included 45/105 (43.8%) Demographic characteristics are summarized in Table 1.

### 3.2. Predisposing Conditions

Among the 105 published cases, a previous history of OPMD or OSCC was documented in 53 patients (50.5%). OPMDs were specifically described in 35 cases, including leukoplakia and its variants in 18 cases, oral lichen planus in 11 cases, verrucous dysplasia or verrucous carcinoma in 3 cases, erythroplakia or erythroleukoplakia in 2 cases, and proliferative verrucous leukoplakia in 1 case. Only four reports explicitly mentioned epithelial dysplasia. In most publications, the precise topographic relationship between the OPMD and the implant site was not detailed. A history of peri-implantitis was recorded in 21 cases (20.0%), and in several instances, peri-implantitis was the initial clinical impression prior to biopsy confirmation.

### 3.3. Risk Factors

Tobacco use was reported in 32 patients (30.5%) and alcohol consumption in 22 (21.0%). Overall, 35 cases (33.3%) presented with at least one traditional carcinogenic exposure, while two-thirds had no identifiable carcinogenic exposure.

### 3.4. Tumor Location and Presentation

The mandible was the most frequently affected site, involved in 91 cases (86.7%), followed by the maxilla in 14 cases (13.3%). Clinically, the majority of tumors presented as exophytic masses (62 cases, 59.0%) or ulcerated lesions (39 cases, 37.1%). Less common presentations included leukoplakia or white plaques (4 cases), peri-implantitis–like inflammation (5 cases), in which lesions were initially misinterpreted as peri-implantitis before histopathologic examination established the diagnosis of OSCC. Additionally, isolated descriptions of swelling, verrucous changes, or granulation tissue were reported.

### 3.5. Implant Technical Characteristics

Reporting of implant-related technical variables was sparse across the included studies. Only a small number of publications explicitly mentioned the implant material, most commonly identifying titanium implants. For example, Brånemark titanium implants [13] and osseointegrated titanium implants [14], with isolated cases consistent with Straumann-type titanium systems [15] or endosseous titanium implants [16]. Hydroxyapatite-coated implants were described only in a few older reports [17,18]. Surface characteristics were reported even less frequently, limited to references to hydroxyapatite coatings [17] and TiUnite anodized surfaces [19]. Prosthetic design was inconsistently documented, typically involving bar-retained overdentures [13,17,20] fixed implant-supported prostheses [21,22] or bridge reconstructions [23]. Limited information was available for loading protocol (e.g., immediate, early, or delayed loading), although some described the duration of implant function prior to diagnosis (approximately ranging from 1 to 8 years). Overall, the absence of standardized reporting prevented systematic evaluation of these variables.

### 3.6. Treatment and Outcomes

The mainstay of treatment was surgical excision with implant removal, combined with or without neck dissection and ± adjuvant radiotherapy. Among cases with follow-up data, local recurrence occurred in 11/105 (10.5%), death in 13/105 (12.4%), and disease-free survival was reported in 26.7%. Follow-up ranged from six months to over 13 years. However, these data were inconsistently provided across case reports. Overall data regarding previous conditions, tumor presentation, and outcomes are detailed in Table 1.

## 4. Discussion

Oral squamous cell carcinoma associated with DI remains a relatively rare clinical finding, and its etiopathogenesis is still not well understood. In 2021, Ramos et al. reported 63 cases of OSCC associated with DI [8]. As of 2025, approximately 105 cases have been published. Although the number of reported cases has increased, the evidence continues to consist almost entirely of isolated case reports and small case series. Because these designs lack control groups, denominators, and standardized reporting, they do not permit reliable quantitative synthesis, statistical comparison, or estimation of relative or attributable risk. Accordingly, the present scoping review is descriptive and should be interpreted as hypothesis-generating rather than inferential.

From a demographic and clinical perspective, the compiled cases generally echoed prior literature, showing a mean age in the late 60 s, slight female predominance, and preference for the posterior mandible [6,8,24]. Given the variability in implant-treated populations, the observed sex distribution likely reflects demographic and treatment-seeking differences rather than biological susceptibility, and thus does not indicate a sex-specific risk.

Clinically, early peri-implant OSCC frequently resembled peri-implantitis or reactive granulation tissue, which contributed to diagnostic delay in several cases [13,24]. Peri-implantitis was described as the initial impression in approximately one-fifth of cases, underscoring the difficulty of distinguishing inflammatory from malignant peri-implant lesions on clinical grounds alone.

Also, over half of the reviewed cases displayed pre-existing mucosal changes such as leukoplakia, erythroplakia, proliferative verrucous leukoplakia, oral lichen planus or cancer. These conditions are characterized by molecular alterations within the epithelium [25]. Thus, this pattern suggests, but does not demonstrate, that many implant-associated OSCCs may arise within previously altered mucosa. Also, examples from various reports such as Gallego et al. [20], Gulati et al. [26], Carini et al. [23], Clapp et al. [17], Galvis et al. [11] and Brabyn et al. [27] describe recurrent or metachronous lesions. However, because of inconsistent reporting, as well as molecular analyses and mucosal mapping were generally not available, the current data do not allow determination of whether these lesions and tumors represent field effects, de novo lesions, recurrences, or second primaries.

Given the increasing prevalence of DIs, estimated to range from 5.7% to 23% by 2026 in the United States, for example [28], OSCC arising in peri-implant tissues may become more frequently encountered. A few studies have mentioned the frequency of OSCC in patients with DI and mostly all coincide in that these cases likely represent a small percentage of all individuals treated with DI [29]. Nevertheless, limited studies have mentioned the role or not of DI in OFC. Moergel et al. studied 2.983 cases that were treated with DI, of which fifteen patients were affected with OSCC. Yet, 9 of those 15 patients were rehabilitated with DI after OSCC diagnosis and treatment [27]. And, although it appeared that the authors did not highlight an association of DI with OFC, it seems that more than 50% of patients with peri-implant OSCC had a history of malignancy. On the other hand, Bhatavadekar et al. mentioned that there is standardized incidence ratio of 0.00017 per 1,000,000 of OSCC cases in patients with DI [15]. Hence, these reports illustrate the wide uncertainty surrounding incidence estimates, driven largely by small case numbers and methodological limitations.

**Figure 2:**
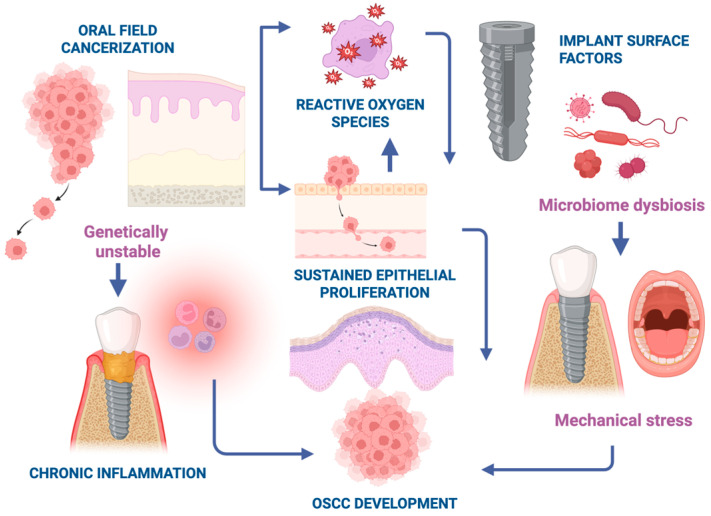
Hypothesized conceptual model of potential factors influencing OSCC occurring adjacent to dental implants. A multifactorial model integrating (1) field-cancerized mucosa with baseline genetic/epigenetic instability, (2) chronic peri-implant inflammation, (3) microbiome dysbiosis, (4) implant surface chemistry and ion release, and (5) mechanical irritation from the prosthesis. These local and systemic influences may interact to create a microenvironment conducive to malignant transformation or recurrence in susceptible mucosa. Created in BioRender. c, m. (2025) https://BioRender.com/3nlcfxb, accessed on 1 November 2020.

While current evidence does not support a causal relationship between DI and oral cancer development, several authors have proposed that local peri-implant factors might act as promoters in genetically susceptible mucosa. As such, in patients without implants, OSCC more commonly involves sites such as the tongue, floor of the mouth, and soft palate. However, the preferential localization of OSCC tumors adjacent to DIs in affected patients also raises important questions about potential mechanistic links. As such, inflammation related to periimplantitis may act as a cofactor in carcinogenesis. Chronic inflammation is known to promote DNA damage through oxidative stress, including the production of reactive oxygen species, which may have pro-oncogenic effects [30]. Also, the surface properties of DIs may further influence carcinogenic potential. Osseointegration depends on cell adhesion, migration, and proliferation. These are properties that could similarly facilitate cancer cell behavior [31]. Cell adhesion to implant surfaces is influenced by both the physical-chemical characteristics of the implant material and host immune response [32]. However, most in vitro studies on implant surfaces have focused on mesenchymal, bone, or stromal cells, not on epithelial or malignant cells [32,33,34,35]. While epithelial attachment is part of the normal healing process [36], the interaction between OSCC cells and implant surfaces remains underexplored. A recent histopathologic study by Verstraeten et al. evaluated OSCC cell invasion at the implant–bone interface and found such infiltration to be uncommon, though the sample size was limited [37]. Additional variables, such as the type of prosthesis used, corrosion of metallic components, and ion release, have also been proposed as contributors to carcinogenesis around DI, but findings remain inconclusive [38]. Nevertheless, these ideas remain theoretical, and the available case-based evidence is insufficient to confirm, quantify, or mechanistically define any such effect (Figure 2).

Similarly, more recent reviews reported that implant-associated OSCC affects patients with prior OPMD or OSCC, without supporting a causal role for implants [7,24]. The present review agrees with these conclusions while offering an updated synthesis through 2025 and a focused consideration of field cancerization and recurrence dynamics. Hence, as observed in this review, a proportion of published implant-associated OSCC cases occurred in patients with clinically altered mucosa or a prior history of OPMD or OSCC. While this pattern suggests possible field cancerization, the available evidence does not allow estimation of risk. Independent of implants, the broader literature on OPMDs and previously treated oral cancer underscores the importance of careful mucosal evaluation and long-term surveillance in individuals with genetically altered epithelium [39,40]. In accordance with these principles, any mucosal area showing leukoplakia, ulceration, erythema, fibrotic scarring, or persistent lesions initially presumed to represent peri-implantitis should undergo prompt histopathologic assessment rather than empirical management, as several case reports describe diagnostic delays when early OSCC was misinterpreted as inflammatory disease. Baseline documentation, including high-quality photographs and appropriate imaging such as panoramic radiography or CBCT, is recommended to record mucosal and osseous conditions prior to treatment [41,42]. Because genetically altered mucosa may remain unstable for many years, structured follow-up is essential, with each visit involving detailed inspection of peri-implant soft tissues and serial clinical documentation as recommended in OPMD and head and neck cancer surveillance guidelines [40,43].

Importantly, the quality of the existing evidence warrants careful consideration. Nearly all available publications consist of isolated case reports, small case series, or retrospective analyses, which are inherently limited by selective reporting, absence of control groups, and lack of standardized diagnostic or follow-up protocols. Key variables, including duration of implant function, severity of peri-implant inflammation, OPMD subtype, dysplasia grade, implant material, prosthetic design, and loading conditions were inconsistently or incompletely reported, resulting in substantial heterogeneity across studies. Recurrence status, time-to-event measures, and survival data were also frequently unavailable, preventing meaningful comparisons with conventional OSCC cohorts or reliable assessment of biological behavior. To date, and to the best of the authors’ knowledge, no prospective or randomized studies have investigated OSCC arising adjacent to dental implants. Consequently, the current evidence base does not support comparative analysis or any valid form of risk quantification or causal inference. Future research should therefore prioritize well-designed multicenter cohorts with standardized baseline documentation and long-term follow-up, complemented by molecular and microbiological investigations capable of distinguishing true recurrences from new primary tumors and clarifying potential pathogenetic pathways.

Despite these constraints, the available cases highlight the need for vigilance when treating patients with field-altered mucosa. Maintaining oncologic safety in implant-rehabilitated patients with field cancerization requires a combination of regular clinical reviews, standardized imaging, and thorough documentation. Close collaboration between surgeons, oral medicine specialists, and pathologists remains essential to ensure early recognition and timely management of malignant or pre-malignant peri-implant lesions.

## 5. Conclusions

In this scoping review, 105 published cases of OSCC arising adjacent to dental implants were identified. Approximately half involved patients with a prior history of OPMD or previous carcinoma, suggesting the presence of field-cancerized mucosa in a substantial subset. The frequent localization of tumors to peri-implant sites also raises hypotheses regarding the potential influence of local factors such as chronic inflammation, epithelial–implant surface interaction, microbiome alterations, and host susceptibility. However, the current evidence does not permit conclusions regarding causality or risk estimation.

From a clinical standpoint, implant rehabilitation in patients with previous OPMD or OSCC should be approached with heightened vigilance. Thorough mucosal assessment, careful patient selection, long-term follow-up, and prompt biopsy of any suspicious peri-implant lesion remain essential to minimize diagnostic delay and improve outcomes.

Future studies should prioritize well-designed prospective multicenter cohorts with standardized baseline documentation, systematic follow-up, and detailed reporting of biological and non-biological variables. Such evidence will be critical for clarifying potential pathogenetic pathways, characterizing risk more accurately, and establishing sound clinical guidelines. Until such evidence becomes available, the patterns observed here should be regarded as exploratory and interpreted with appropriate caution.

## Figures and Tables

**Table 1:** Clinical findings of cases of oral squamous cell carcinoma associated with osseointegrated dental implants.

	Number
	**Reported cases**
**Age (average)**	66.8
**Range**	40–90
F:M ratio	1.33:1
**Site**	
Mandible	91/105 (86.7%)
Maxilla	14/105 (13.3%)
**Previous history of OPMD/cancer ^a^**	53/105 (50%)
**Previous history of peri-implantitis**	21/105 (20.0%)
**Clinical presentation**	
Exophytic mass/mass	62/105 (59.0%)
Granulation tissue	1/105 (1.0%)
Leukoplakia/white plaque	4/105 (3.8%)
Ulcer/ulcerated lesion	39/105 (37.1%)
Peri-implantitis	5/105 (4.7%)
Normal mucosa	1/105 (1.0%)
Swelling	3/105 (2.8%)
Verrucous lesion	2/105 (1.9%)
**Outcome ^a^**	
Recurrence	11
Metastases	1
New primary tumors	4
Death	13

^a^ Outcome percentages are not reported because outcome data were mostly inconsistently provided across case reports.

## Data Availability

No new data were created or analysed in this study. Data sharing is not applicable to this article.

## Supplementary Materials

The following supporting information can be downloaded at: https://www.mdpi.com/article/10.3390/cancers18010017/s1. Table S1. Summary of published literature about oral squamous cell carcinoma associated with osseointegrated dental implants. References [44,45,46,47,48,49,50,51,52,53,54,55,56,57,58,59,60,61,62,63,64,65,66] are cited in Supplementary Materials.
