# Supplementary material for: Oral Squamous Cell Carcinoma Associated with Dental Implants: A Literature Review with Focus on Field-Cancerized Mucosa
Source: Cancers (Basel). 2025 Dec 19;18(1):17. doi: 10.3390/cancers18010017 (PMC12784837; doi:10.3390/cancers18010017)
Supplement: Supplementary file 1 [file cancers-18-00017-s001.zip › cancers-4002620-supplementary.pdf]

Table S1. Summary of published literature about oral squamous cell carcinoma associated with osseointegrated dental implants.

	Year	Author	Age	M/F	Previous history of OSSC/ OPMD	Previous history of Peri-implantitis	Risk Factors	Location	Clinical presentation	Outcome	Follow up
1	1983	Friedman and Vernon[44]	65	M	N/A	Yes	Smoking	Mandible	Ulcerated mass	N/A	N/A
2	1996	Clapp et al.[17]	65	F	None	N/A	None	Mandible	Ulceration	Recurrence	36 months
3	1996	Clapp et al.[17]	79	M	None	N/A	Alcohol, smoking	Mandible	Ulceration	Recurrence	N/A
4	1996	Clapp et al.[17]	90	F	OED/ OSCC	N/A	Ex-smoker, Alcohol	Mandible	Ulceration	None	12 months
5	1997	Moxley et al.[45]	74	F	Verrucous carcinoma	Yes	Alcohol	Mandible	Exophytic mass	N/A	N/A
6	2001	Block and Scheufler[18]	72	M	Verrucous carcinomas/ leukoplakia	None	Ex-smoker	Mandible	N/A	None	18 months
7	2004	Schaw et al.[21]	67	M	OSCC	Yes	N/A	Mandible	Exophytic mass	N/A	N/A
8	2004	Schaw et al.[21]	69	F	OSCC/verrucous leukoplakia	None	N/A	Mandible	Exophytic mass	N/A	N/A
9	2006	Czerminski et al.[14]	52	F	OLP	N/A	Smoking	Mandible	Ulcer/ exophytic mass	None	18 months
10	2006	Czerminski et al.[14]	80	M	OSCC	N/A	N/R	Mandible	Ulcerated exophytic mass	Death	Less than 12 months
11	2007	Abu El-Naaj et al.[46]	70	M	None	None	None	Mandible	Exophytic mass	N/A	N/A
12	2007	Abu El-Naaj et al.[46]	72	F	OLP	None	Smoking	Mandible	Ulcer	N/A	N/A
13	2008	Chimenos-Küstner et al.[47]	62	F	N/A	N/A	Ex-smoker/ Alcohol use	Mandible	Exophytic mass	N/A	N/A
14	2008	Eguiar et al.[22]	76	M	N/A	Yes	None	Mandible	Ulcerated exophytic mass	N/A	N/A
15	2008	Gallego et al.[20]	81	F	OLP/OSCC	N/A	None	Mandible	Exophytic mass	Recurrence	12 months
16	2008	Kwok et al.[48]	62	M	N/A	N/A	Alcohol, smoking	Mandible	Non-healing ulcer	None	36 months

17	2008	Kwok et al.[48]	71	M	None	Yes	Alcohol use, smoking	Mandible	Peri-implantitis	Death	N/A
18	2008	Kwok et al.[48]	67	F	OSCC	No	Ex-smoker/ alcohol use	Mandible	Granulation tissue	None	24 months
19	2008	Schache et al.[49]	77	M	None	None	None	Mandible	Exophytic mass	N/A	N/A
20	2009	Gallego et al.[50]	70	F	None	None	None	Mandible	Traumatic ulcer	None	12 months
21	2009	Gulati et al.[26]	62	F	OSCC	Yes	Smoking	Mandible	Leukoplakia	Recurrences/death	24 months+
22	2010	De Ceulaer et al.[51]	77	F	OSCC	Yes	N/A	Mandible	N/A	None	6 months
23	2010	De Ceular et al.[51]	71	M	OSCC	No	N/A	Mandible	Swelling	Death	N/A
24	2010	De Ceulaer et al.[51]	62	F	OSCC	Yes	N/A	Mandible	N/A	N/A	N/A
25	2010	Meijer et al.[19]	65	F	OSCC	N/A	N/A	Mandible	Exophytic mass	None	36 months
26	2011	Moshref et al.[52]	67	F	None	Yes	None	Mandible	Exophytic mass	N/A	N/A
27	2012	Bhatavadekar.[15]	54	M	None	No	None	Maxilla	Ulcer	N/A	N/A
28	2012	Carini et al.[23]	70	F	Leukoplakia /carcinoma	N/A	N/A	Mandible	Ulcer	Recurrence	12 months+
29	2013	Marini et al.[16]	51	F	OLP	None	None	Mandible	Exophytic mass	None	60 months
30	2014	Moergel et al.[29]	63	F	Leukoplakia /carcinoma	N/A	Smoking and/or alcohol	Mandible	Exophytic mass	N/A	80 months
31	2014	Moergel et al.[29]	70	F	Leukoplakia /carcinoma	N/A	Smoking	Mandible	Exophytic mass	N/A	14 months
32	2014	Moergel et al.[29]	72	M	Leukoplakia /carcinoma	N/A	None	Mandible	Exophytic mass	N/A	N/A
33	2014	Moergel et al.[29]	57	M	Leukoplakia /carcinoma	N/A	Smoking/ alcohol	Mandible	Peri-implantitis	N/A	N/A
34	2014	Moergel et al.[29]	72	M	Leukoplakia	N/A	N/A	Mandible	Exophytic mass	N/A	120 months
35	2014	Moergel et al.[29]	54	F	OLP	N/A	Ex-smoker	Mandible	Exophytic mass		N/A
36	2014	Moergel et al.[29]	47	M	Unspecified malignancy	N/A	Smoking/ alcohol	Mandible	Ulcer	N/A	N/A
37	2014	Moergel et al.[29]	88	M	Leukoplakia	N/A	None	Mandible	Ulcer	N/A	N/A
38	2014	Moergel et al.[29]	42	F	Leukoplakia /carcinoma	N/A	N/A	Mandible	Ulcer	Death	N/A

39	2014	Moergel et al.[29]	59	F	Leukoplakia /carcinoma	N/A	N/A	Mandible	Ulcer	N/A	N/A
40	2014	Moergel et al.[29]	73	M	Leukoplakia /erythroplakia/carcinoma	N/A	Ex-smoking/ alcohol	Maxilla	Exophytic mass	N/A	3 months
41	2014	Moergel et al.[29]	77	M	Leukoplakia	N/A	Smoking/ alcohol	Mandible	Exophytic mass	N/A	N/A
42	2014	Moergel et al.[29]	68	F	Leukoplakia	N/A	Smoking/ alcohol	Mandible	Exophytic mass	N/A	129 months
43	2014	Moergel et al.[29]	69	F	OLP	N/A	None	Mandible	Exophytic mass	Death	N/A
44	2014	Moergel et al.[29]	80	F	Leukoplakia /OLP	N/A	Smoking/ alcohol	Mandible	Exophytic mass	Death	N/A
45	2015	Chainani-Wu et al.[53]	60	F	Erythroleukoplakia, OED	Yes	None	Mandible	Normal mucosa	2nd primary tumor	24 months
46	2015	Nairai et a.[54]	58	F	OSCC	N/A	Smoking/ alcohol	Mandible	White mass	None	24 months
47	2016	Bhandari et al.[55]	76	F	N/A	Yes	None	Maxilla	Peri-implantitis	None	11 months
48	2016	Raiser et al.[56]	55	F	OLP	None	N/A	Mandible	Exophytic mass	None	86 months
49	2016	Raiser et al.[56]	72	M	OLP	None	N/A	Maxilla	Exophytic mass	None	36 months
50	2017	Noguchi et al.[57]	65	F	None	Yes	Alcohol	Mandible	Exophytic mass	None	12 months
51	2017	Ito et al.[10]	62	M	None	Yes	Alcohol, Ex-smoker	Maxilla	Peri-implantitis	None	24 months
52	2017	Kaplan et al.[58]	73	F	None	N/A	N/A	Maxilla	Ulcerated mass	Death	N/A
53	2017	Kaplan et al.[58]	71	M	Leukoplakia /OLP	N/A	N/A	Mandible	Mass	Recurrence	2 months
54	2017	Kaplan et al.[58]	44	M	None	N/A	N/A	Maxilla	Mass	None	24 months
55	2017	Kaplan et al.[58]	59	F	OLP	N/A	N/A	Mandible	Mass	None	18 months
56	2017	Kaplan et al.[58]	77	F	PVL/OSCC	N/A	N/A	Mandible	Mass	Recurrence	16 months
57	2017	Norton.[59]		F	N/A	Yes	N/A	Mandible	Peri-implantitis	Multiple metastases/ death	60 months
58	2018	Carreira-Nestares et al.[60]	85	F	N/A	N/A	Smoking and or alcohol	Mandible	Ulcer	N/A	N/A
59	2018	Oh et al.[61]	43	M	N/A	N/A	None	Mandible	Ulcer	N/A	N/A
60	2018	Brabyn et al.[27]	79	F	None	N/A	None	Mandible	N/A	Death	48 months

61	2018	Brabyn et al.[27]	71	M	OSCC	N/A	Ex-smoker/alcohol use	Mandible	Exophytic mass	Recurrence, second and third primary tumor	72 months
62	2018	Brabyn et al.[27]	62	F	OSCC, verrucous dysplasia	N/A	Ex-smoker	Maxilla	Exophytic, verrucous mass	Recurrence and second primary tumors	N/A
63	2018	Brabyn et al.[27]	74	F	Leukoplakia, OED and OSCC	N/A	None	Mandible	Exophytic mass	Recurrence, multiple second primary tumors, metastases and death	156 months
64	2018	Brabyn et al.[27]	64	F	OSCC	N/A	None	Mandible	N/A	None	N/A
65	2018	Brabyn et al.[27]	51	M	None	N/A	Smoking	Mandible	Ulcerated lesion	Death	N/A
66	2019	Malthièry et al.[62]	73	M	N/A	Yes	None	Mandible	Exophytic mass	None	48 months
67	2019	Ngouchi et al.[63]	78	F	OLP	None	Alcohol use	Mandible	Exophytic mass	None	48 months
68	2019	Coopman et al.[13]	76	F	None	Yes	None	Mandible	Exophytic mass	N/A	NA
69	2020	Granados et al.[64]	83	M	OSCC	N/A	N/A	Mandible	Ulcer	N/A	N/A
70	2020	Granados et al.[64]	60	M	N/A	N/A	N/A	Mandible	Verrucous lesion	N/A	N/A
71	2020	Granados et al.[64]	54	F	None	N/A	N/A	Mandible	N/A	N/A	N/A
72	2020	Granados et al.[64]	64	M	None	N/A	N/A	Mandible	N/A	Death	6 months
73	2022	Galvis et al.[11]	85	M	OSCC	Yes	N/A	Mandible	Exophytic mass	Recurrences	N/A
74	2022	Galvis et al.[11]	81	F	OSCC	Yes	N/A	Mandible	Ulcer	Recurrence	14 months
75	2022	Galvis et al.[11]	N/A	F	None	Yes	N/A	Mandible	Swelling/ulcer	None	33 months
76	2022	Galvis et al.[11]	61	M	None	Yes	Ex-smoker	Mandible	Swelling/ulcer	None	62 months
77	2022	Galvis et al.[11]	77	F	None	None	N/A	Mandible	White plaque	None	96 months
78	2022	Galvis et al.[11]	74	M	None	None	Smoker	N/A	Ulcer	None	72 months
79	2022	Galvis et al.[11]	64	F	None	None	None	Mandible	Exophytic mass	N/A	N/A

80	2022	Galvis et al.[11]	65	F	None	None	None	Mandible	Ulcer	Recurrences (2)	26 months
81	2022	Galvis et al.[11]	84	F	OSCC	None		Mandible	Exophytic mass	None	50 months
82	2022	Galvis et al.[11]	68	F	Leukoplakia	None	N/A	Mandible	Leukoplakia	None	12
83	2022	Galvis et al.[11]	90	F	None	None	N/A	Mandible	White plaque	None	12
84	2022	Galvis et al.[11]	73	F	OSCC	N/A	N/A	Mandible	N/A	None	1
85	2022	Park et al.[65]	57	M	N/	None	Smoking	Maxilla	Ulcerated mass	N/A	N/A
86	2024	Seo M et al.[66]	44	M	None	N/A	None	Mandible	Exophytic mass	N/A	70 months
87	2024	Seo M et al.[66]	48	F	OSCC	N/A	None	Mandible	Exophytic mass	N/A	70 months
88	2024	Seo M et al.[66]	54	M	None	N/A	Smoking/ alcohol	Mandible	Exophytic mass	N/A	70 months
89	2024	Seo M et al.[66]	56	F	None	N/A	None	Mandible	Exophytic/ulcerated mass	N/A	70 months
90	2024	Seo M et al.[66]	72	M	None	N/A	None	Mandible	Exophytic/ulcerated mass	N/A	70 months
91	2024	Seo M et al.[66]	61	M	None	N/A	None	Mandible	Ulcerated	N/A	70 months
92	2024	Seo M et al.[66]	69	F	None	N/A	None	Mandible	Exophytic/ulcerated mass	N/A	70 months
93	2024	Seo M et al.[66]	68	F	None	N/A	None	Mandible	Ulcerated	N/A	70 months
94	2024	Seo M et al.[66]	61	M	None	N/A	Smoking/ alcohol	Maxilla	Exophytic/ulcerated mass	N/A	70 months
95	2024	Seo M et al.[66]	59	M	None	N/A	None	Mandible	Exophytic/ulcerated mass	N/A	70 months
96	2024	Seo M et al.[66]	63	M	None	N/A	None	Mandible	Exophytic/ulcerated mass	N/A	70 months
97	2024	Seo M et al.[66]	64	F	None	N/A	None	Maxilla	Exophytic/ulcerated mass	N/A	70 months
98	2024	Seo M et al.[66]	76	F	OLP	N/A	None	Mandible	Exophytic mass	N/A	70 months
99	2024	Seo M et al.[66]	58	M	OSCC	N/A	None	Mandible	Exophytic/ulcerated mass	N/A	70 months
100	2024	Seo M et al.[66]	63	M	None	N/A	Smoking	Maxilla	Exophytic mass	N/A	70 months
101	2024	Seo M et al.[66]	58	F	None	N/A	None	Mandible	Exophytic mass	N/A	70 months
102	2024	Seo M et al.[66]	40	F	None	N/A	None	Mandible	Exophytic mass	N/A	70 months
103	2024	Seo M et al.[66]	71	M	None	N/A	None	Mandible	Exophytic/ulcerated mass	N/A	70 months
104	2024	Seo M et al.[66]	63	M	None	N/A	None	Maxilla	Exophytic mass	N/A	70 months

105	2024	Seo M et al.[66]	70	M	OSCC	N/A	None	Mandible	Exophytic/ulcerated mass	N/A	70 months

N/A: data not available; M: Male; F: Female; OED, oral epithelial dysplasia; PVL: Proliferative verrucous leukoplakia.
